# Onion Skin or Common Drive?

**DOI:** 10.3389/fncel.2017.00002

**Published:** 2017-01-19

**Authors:** Maria Piotrkiewicz, Kemal S. Türker

**Affiliations:** ^1^Department of Engineering of Nervous and Muscular System, Nalecz Institute of Biocybernetics and Biomedical Engineering, Polish Academy of SciencesWarsaw, Poland; ^2^Laboratory of Neuromuscular Research, Koç University School of MedicineIstanbul, Turkey

**Keywords:** firing patterns, human motoneuron, motor unit, surface EMG decomposition, force gradation

A motor unit (MU), which consists of a single motoneuron (MN) and the muscle fibers it innervates, is an essential element of the motor control system. The knowledge in this field, collected over decades, is based on research conducted on both animal and human model systems. Experiments on animals allow direct measurement of the MN characteristics and the contractile properties of the muscle fibers it innervates. Thus, all essential information on the properties of the basic elements of the motor system was obtained from animal studies. In contrast, experiments performed on human subjects, which for obvious reasons rely on indirect methods, study intact MUs in their physiological environment during voluntary contractions. Both types of model systems are complementary, because each system collects information that is difficult or impossible to obtain in the other model system. Not all results from animal studies can be verified in human experiments, but we see no reason to think that the basic principles of motor control are different in animals and human subjects.

The control of muscle force involves two essential mechanisms: MU recruitment and rate coding. Earlier studies in motor control provide detailed information on both mechanisms. These studies show that MNs are recruited in an orderly fashion from smallest to largest, as seen in animal muscles (Henneman, 1957; Henneman et al., 1974), and in human muscles (Milner-Brown et al., 1973a,b). For an in depth review on the different aspects of orderly recruitment of MN, see Bawa et al. (2014).

In cats, motor units were classified by Burke et al. (1973) on the basis of their twitch contraction time into: (i) slow (S), innervated by the smallest MNs, (ii) fast fatigable (FF), innervated by largest MNs, and (iii) fast resistant to fatigue (FR), controlled by MNs of intermediate size. Slow MUs, recruited at the lowest force levels, are practically not fatigable and may function for several hours, while FF MUs are recruited at the highest force levels for short amounts of time. In human muscles, the results concerning MU recruitment order were inconclusive. In studies by Macefield et al. (1996) and Bigland-Ritchie et al. (1998), no correlation between MU size and contraction speed was found, whereas Milner-Brown et al. (1973a) reported that the larger, higher-threshold MUs tend to have shorter contraction times than the smaller, lower-threshold ones. Since the former studies did not measure recruitment threshold, we may assume that human MUs recruited close to maximal voluntary contraction (MVC) are faster than the low-threshold ones.

The force-rate relationship of a MU depends on its contraction time. The steepest part of the relationship corresponds to the optimal working range of the MU (Kernell, 1983, 2006; Piotrkiewicz and Celichowski, 2007). As seen in Figure 1A, the MU reaches its maximum tetanic force at a certain firing rate which is inversely dependent on its twitch duration (Bellemare et al., 1983; Kernell, 1983, 2003). Increasing the firing rate above this rate is not compatible with optimal contraction control (Bigland-Ritchie and Woods, 1984). Therefore, the rate saturation observed by many researchers in low-threshold human MUs is not surprising (Bigland and Lippold, 1954; Gydikov and Kosarov, 1974; Monster and Chan, 1977; Bellemare et al., 1983; Moritz et al., 2005; Bailey et al., 2007; Fuglevand et al., 2015).

**Figure 1:**
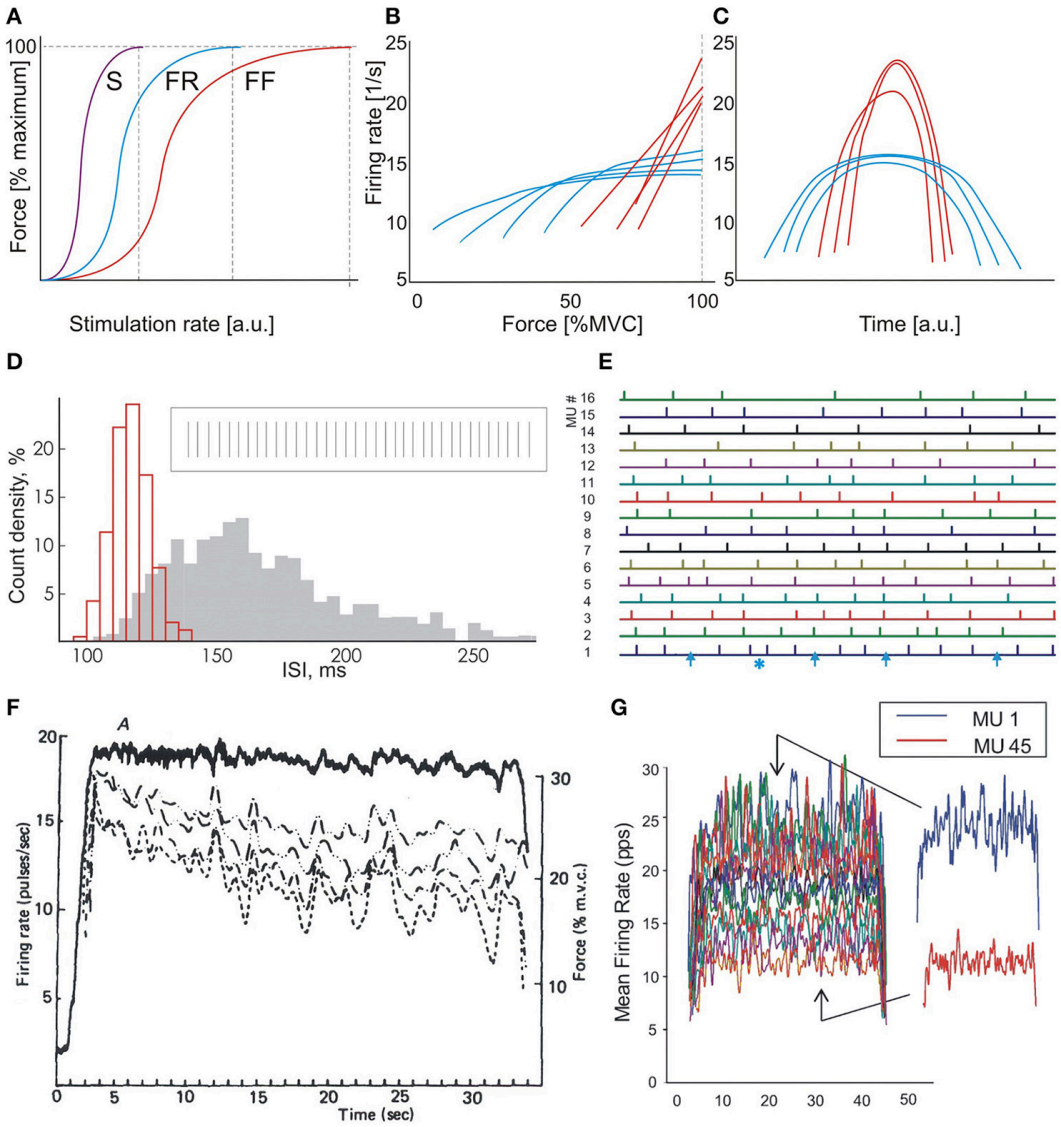
**Motor unit behavior in isometric contractions. (A)** Force-rate characteristics of three MU types: slow (S), fast fatigue resistant (FR), and fast fatigable (FF). Vertical dashed lines indicate rates, at which full tetani are obtained. **(B)** Dependencies of firing rate on contraction force: blue lines, low-threshold MUs, red lines, high-threshold MUs. Scheme based on the results of Gydikov and Kosarov (1974). **(C)** “Reversed onion skin” scheme. Higher-threshold MUs (red lines) fire with rates exceeding those of lower-threshold units (blue lines), which exhibit regular “onion skin” pattern. Scheme based on the results of Oya et al. (2009). **(D)** Histograms of two MUs firing with high (red) and low (gray) rate, respectively. Insert: fragment of discharge sequence of an MU firing with high rate. Modified Figure 7 from Piotrkiewicz et al. (2001). **(E)** Fragment of the bar raster from Figure 4 of Nawab et al. (2010). Bars represent discharges of 16 MUs, numbered in the order of recruitment thresholds. Arrows at the lowest trace indicate very long intervals; asterisk indicates a very short interval. **(F)** Firing-rate records of four concurrently active motor units (dashed lines) and the force output (continuous line) recorded during a constant-force muscle isometric contraction (Figure 1 from De Luca et al., 1982a). **(G)** Mean firing rates of 45 MUs concurrently active at 35% contraction (Figure 9 from De Luca and Contessa, 2012). The permissions from Wiley and Sons, Elsevier and Nalecz Institute of Biocybernetics and Biomedical Engineering are kindly acknowledged.

When the muscle force is increased, higher-threshold MUs are gradually recruited, and lower-threshold MUs increase their firing rates. Fast MUs, recruited at high force levels, have their optimal working range and full tetanus shifted toward higher rates. Thus, it may be expected that these MUs would fire with rates exceeding those achieved by low-threshold ones. In fact, results from human experiments are in line with this expectation. Gydikov and Kosarov (1974) observed two MU types, which differed by their discharge properties. The firing rates of the lower-threshold MUs initially increased with muscle force and saturated at higher forces. The higher-threshold MUs increased their firing rates linearly up to maximal voluntary force, reaching rates higher than those of lower-threshold MUs (see Figure 1B). Similar results were reported by other researchers (Bigland and Lippold, 1954; Monster and Chan, 1977; Bellemare et al., 1983; Bigland-Ritchie et al., 1983; Moritz et al., 2005; Bailey et al., 2007).

The majority of information on rate coding in humans was collected at low force levels. These studies showed that the firing rates of newly recruited MUs are lower than those of the earlier recruited ones (Person and Kudina, 1972; Tanji and Kato, 1973). The plot of mean MU firing rates vs. time during contractions of a triangular force profile has a typical appearance of an onion skin (Figure 1C, blue curves). Thus, this phenomenon was named as the *onion skin* by De Luca et al. (1982a, Figure 1C).

However, the above presented evidence on the behavior of lower- and higher-threshold MUs indicates that the firing rates of the latter can be greater than those of the former at the highest force levels. Thus, the collective MU behavior at highest force levels should be closer to another pattern (Figure 1C). This behavior was denominated by Hu et al. (2014) as *reversed onion skin*. Such a pattern was indeed observed by Oya et al. (2009).

All results from research on human muscle force control, summarized above, are now questioned by a group of scientists led by Carlo De Luca. They propose the onion skin scheme as the basic principle underlying human motor control (e.g., De Luca and Contessa, 2012, 2015). This view is based on results produced by the computer system for MU recording and decomposition from the surface electromyogram picked up by a sensor composed from 5 small electrodes. The authors claim that this system can identify more than 50 single MU potential (MUP) trains recorded during a MVC with more than 90% accuracy.

To validate the high performance of their system, De Luca and colleagues applied two methods (De Luca and Hostage, 2010; Nawab et al., 2010): (i) two-sensor and (ii) “reconstruct-and test” or “Decompose-Synthesize-Decompose-Compare” (DSDC). The latter composes synthetic EMG from the MUP templates and firing instances derived from decomposition. The final result is obtained by adding noise with a power similar to that of the residual in the first decomposition. The synthesized signal is then decomposed and the two decompositions are compared to estimate their accuracy. This method was criticized by Farina and Enoka (2011), who stated that “Contrary to the two-sensor method, the reconstruct-and-test procedure is biased in that the signal used in the second decomposition *depends* on the result of the first decomposition and may lead to an estimation of 100% accuracy for a train of action potentials, even when a substantial number of discharge times are not identified.” The two-sensor method also does not take unidentified potentials into account and even the authors admit that “the degree of agreement between two imperfect decompositions does not offer sufficient proof about the degree of accuracy of either one” (Nawab et al., 2010).

The number of unidentified potentials increases with force level due to increasing phase cancelation (e.g., Tucker and Türker, 2005), which happens more often with surface electrodes than with intramuscular ones. The published papers of De Luca and colleagues contain clear evidence that this number can be quite substantial, especially for low-threshold MUs. These MUs are firing at high rates, when their interspike interval distribution is normal and narrow (Figure 1D) and their discharge is expected to be regular. However, specifically referring to Figure 4 from Nawab et al. (2010), trace #1, representing the lowest-threshold MU, contains 4 intervals that are approximately twice as long as the others (Figure 1E, blue arrows). These long intervals are probably due to missed MUPs. The discharge regularity is also disturbed by a short interval, which raises further concerns regarding the accuracy of decomposition.

Such decomposition errors may be responsible for the disappearance of the correlation between fluctuations of the firing rates of simultaneously firing MUs that can be seen in some of De Luca et al.'s results (Figure 1F). This common fluctuation phenomenon was observed in earlier studies (e.g., Person and Kudina, 1972) and was named common drive by De Luca et al.(1982b), De Luca and Erim (1994). Common drive was proposed to be the general strategy for increasing muscle force by the central nervous system and the authors of this opinion find this hypothesis justified. However, in recent literature based on the 5-pin surface EMG decomposition system (e.g., De Luca and Contessa, 2012), no sign of this collective behavior can be observed (Figure 1G).

Given the decomposition errors indicated above, we conclude that results of decomposition performed at high muscle forces have to be treated with caution, especially when they contradict the knowledge collected so far.

The 5-pin surface electrode system can produce reliable results, especially when it is used by researchers who do not aim for analysis of all MUs firing at 100% MVC, and/or by those who are able to develop reliable procedures for detection and rejection of poorly decomposed MUP trains. For example, Suresh et al. (2014) used this system at muscle contractions of the lowest possible force, at which the potentials of only one MU could be reliably distinguished. Hu et al. (2013) combined the spike-triggered averaging of surface EMG with DSDC validation, which allowed them to detect unreliable MU traces. They observed the onion skin pattern for contractions up to 50% MVC, which did not contradict the results of Gydikov and Kosarov (1974).

There are other researchers, who investigate collective firing behavior of MUs by means of high-density surface electrode arrays. They also rely on sophisticated decomposition algorithms (e.g., Holobar et al., 2010; Yavuz et al., 2015; Negro et al., 2016). Validation is performed after every decomposition, and MUP trains that do not fulfill reliability criteria are excluded from further analysis.

We believe that there is plenty to discover when it concerns collective MU behavior at high force contraction levels. Selective surface electrode systems have many advantages and may be very helpful in such investigations. However, potential users should be aware of the possible flaws of these systems and should be especially careful when formulating conclusions that contradict all previously known research.

It is our opinion that the common drive phenomenon indeed belongs to those strategies of the motor control system that are likely to be functional at any contraction level, whereas the onion skin phenomenon still needs reliable testing at highest force levels.

## Author contributions

MP conceived the idea and wrote the first draft. Both MP and KT discussed and corrected the manuscript until the final version was ready.

## Funding

Both authors were supported by their employing institutions.

### Conflict of interest statement

The authors declare that the research was conducted in the absence of any commercial or financial relationships that could be construed as a potential conflict of interest.
